# Sex-differential association of suicide attempts with thyroid dysfunction in first-episode and drug-naïve young major depressive disorder patients with comorbid anxiety

**DOI:** 10.1038/s41598-023-40948-2

**Published:** 2023-08-22

**Authors:** Gang Ye, Ying Yuan, Zhe Li, Yan Yue, Yuxuan Wu, Ruchang Yang, Haitao Wang, Siqi Wu, Yue Zhou, Xueli Zhao, Xiaoli Lv, Nian Yuan, Ronghua Li, Guangya Zhang, Xiangdong Du, Xiangyang Zhang

**Affiliations:** 1 Suzhou Medical College of Soochow University, Suzhou, People’s Republic of China; 2 Suzhou Guangji Hospital, The Affiliated Guangji Hospital of Soochow University, No. 11 Guangqian Road, Suzhou, 215137 Jiangsu Province People’s Republic of China; 3 School of Psychology and Mental Health, North China University of Science and Technology, Tangshan, People’s Republic of China; 4 Xuzhou Medical University, Xuzhou, People’s Republic of China; 5 CAS Key Laboratory of Mental Health, Institute of Psychology, Chinese Academy of Sciences, Beijing, People’s Republic of China

**Keywords:** Depression, Thyroid hormones, Risk factors

## Abstract

This study aimed to explore sex differences in the relationship between thyroid function indicators and suicide attempts in first-episode and drug-naïve young major depressive disorder (MDD) patients with comorbid anxiety (MDA). A total of 917 MDD patients (aged 18–35 years) were recruited. The Hamilton depression rating scale (HAMD-17), Hamilton anxiety rating scale (HAMA), positive and negative syndrome scale (PANSS) positive subscale and clinical global impression of severity scale (CGI-S) were used. 467 patients were classified as MDA. The prevalence of suicide attempts was 31.3% in MDA patients, which was significantly higher than that (7.3%) in MDD patients without anxiety. Compared with MDA patients without suicide attempts, MDA patients with suicide attempts were older, had a later age of onset, higher HAMD-17, HAMA, and PANSS positive symptom subscale scores, as well as higher TSH, TgAb and TPOAb levels. For male patients, TSH and TPOAb levels were independently associated with suicide attempts. For female patients, HAMA, PANSS positive symptom scores, CGI-S score and TPOAb levels were independently associated with suicide attempts. Our results suggest that the indicators of thyroid function which can predict suicide attempts in male and female MDA patients have sex differences.

## Introduction

Suicide is a major public health problem worldwide and is considered to be the second leading cause of death among adolescents and young adults^1^. The incidence of suicide attempts is highest among young adults across different countries^2^ and there is also a peak in the 15–34 years olds in China^3^. A large body of evidence supports the link between major depressive disorder (MDD) and suicide in the general population; however, there is a lack of research in the field of young adult patients with MDD. Actually, the most likely period for the first episode of MDD is from mid-adolescence to mid-40s, with almost 40% experiencing their first depressive episode before the age of 20 years^4^. There is a need to better understand suicide in younger MDD patients.

MDD is a heterogeneous disorder with different clinical features, such as anxiety. In China, the prevalence of comorbid anxiety in MDD patients is high, but the detection rate is low^5^. Anxiety has been confirmed to increase the severity of MDD and make it more difficult to be treated^6^. However, whether comorbid anxiety increases the risk of suicide attempts in MDD patients remains controversial. Most of previous studies support anxiety as a risk factor for suicide, although more studies are needed before this association can be validated^7,8^. We speculate that anxiety may have different effects on suicidality in different subgroups such as young MDD patients. However, to date, there is a lack of studies in this field.

Another important question is what causes suicidality in MDD patients, especially in major depression combined with anxiety (MDA) patients. Several studies have found that thyroid dysfunction plays an important role in suicide. A recent meta-analysis shows that patients with suicidal behavior have significantly lower FT3 and TT4 levels compared to patients without suicidal behavior^9^. Meanwhile, thyroid dysfunction has been proved to be associated with mood disorders including depression and anxiety. Previous studies have also demonstrated a link between thyroid hormone levels and anxiety-depression in hypothyroidism or hyperthyroidism patients^10^. Moreover, the association between depression or anxiety and thyroid function is also affected by sex. For instance, Gorkhali et al.^11^ have found that depression and anxiety are highly prevalent in patients with thyroid disorders, especially in women. Sex differences can also be found in the correlation between suicide and depression^12,13^. Previous studies have shown that female patients have a significantly higher rate of suicide attempts than male patients^14^, while the opposite is true for complete suicides^15^. However, it remains unclear whether there are sex differences both in the incidence of suicide attempts and in the association with thyroid hormone levels in young MDD patients with comorbid anxiety (MDA).

Therefore, the main aim of this cross-sectional study was to explore the prevalence of suicide attempts and the association with thyroid function indicators, as well as their sex differences in first-episode and drug-naïve young major depressive disorder patients with comorbid anxiety.

## Methods

### Ethics statement

This study was conducted in the psychiatric outpatient department of a general hospital in Taiyuan, Shanxi province, China. The protocol for this study was approved by the Institutional Review Board, the First Clinical Medical College, Shanxi Medical University (No. 2016-Y27). Written informed consent was obtained after a complete description of the study protocol and procedures was given to each participant by the psychiatrist or research coordinator. The study was performed in accordance with relevant guidelines regulated in the above-mentioned Institutional Review Board.

### Subjects

A total of 917 young adult patients with MDD (male/female = 351/566) were recruited. The following inclusion criteria were applied: (1) 18–35 years old, Han Chinese; (2) diagnosed of MDD according to the Diagnostic and Statistical Manual of Mental Disorders, Fourth Edition (DSM-IV); (3) the first episode; (4) no history of any antidepressant or antipsychotic treatment; (5) able to participate in clinical assessment.

The Hamilton Anxiety Rating Scale (HAMA) was used to assess anxiety symptoms. All subjects were divided into two groups based on their HAMA scores. According to the scoring rules of the Chinese version of the HAMA, a total score of ≥ 29 indicates severe anxiety, ≥ 21 indicates obvious anxiety, and ≥ 14 indicates some anxiety, ≥ 7 indicates possible anxiety and < 7 indicates no anxiety^16^. According to this rule, subjects with scores ≥ 21 were classified as MDD with comorbid anxiety (MDA) group, and other subjects were classified as MDD without anxiety (MDNA) group. To our knowledge, many previous studies also adopted these scoring rules to assess anxiety symptoms^17–19^.

All participants received a comprehensive physical examination, medical history assessment and laboratory tests to determine that they were in good physical health. Participants were excluded if they had any medication intake that could affect thyroid function. Other exclusion criteria included comorbid any other major Axis I disorder, drug or alcohol abuse, neurodegenerative and neurological disorders, and pregnant or lactating women. Patients who were unable to sign an informed consent form were also excluded.

All participants were diagnosed as MDD by two trained clinical psychiatrists based on the Diagnostic Interview of the Chinese version of Structured Clinical Interview for DSM-IV (SCID). Comorbid mental disorders were determined mainly on the basis of psychiatric history, mental examination, and self-reports. At the same time, the psychiatrists who participated in this study were trained in the assessment of the Hamilton Depression Inventory scale (HAMD-17), the HAMA scale, and the PANSS Positive Symptom Subscale before conducting the clinical assessment. For all three of these clinical assessment scales, the internal consistency of the independent blind raters was above 0.8.

### Clinical measurements

Demographic and clinical data were collected for each subject using a detailed questionnaire. For the purpose of this study, suicide attempts were defined as self-injurious behavior, with an intent to end one's life, but not resulting in death. All subjects and/or their family members were asked, “During your (or the patient's) lifetime, have you (he or she) ever attempted suicide?” If they answered “yes”, these patients were considered to be suicide attempters. For patients with suicide attempts, further details were collected, including: the number of suicide attempts, the exact date, and the method of the suicide attempt.

The Hamilton depression inventory and the Hamilton anxiety inventory were used to assess depressive and anxiety symptoms, respectively. The PANSS Positive Symptom Subscale was used to assess psychotic symptoms. The clinical global impression of severity scale (CGI-S) was used to assess the severity of the disease. The scales adopted in this study have been shown to have good reliability and validity in previous studies and have been widely used in China. The Cronbach's alpha coefficient for the HAMA is 0.93, and the Cronbach's alpha coefficient for the HAMD-17 after localization in China is 0.88–0.99^16^. The Cronbach's alpha coefficient for the PANSS is 0.928 and the intra-class coefficient is 0.878 (95% CI 0.79–0.92)^20^. Studies which have tested the reliability of the CGI-S scale demonstrated good inter-rater reliability for it^21^.

### Measurement of thyroid function indicators

On the day of clinical data collection, blood samples were collected in the morning (between 6 and 8 am) after an overnight fast. All samples were immediately sent to the hospital's laboratory center and serum levels of thyroid stimulating hormone (TSH), free triiodothyronine (FT3), free thyroxine (FT4), antithyroglobulinand (TgAb), and thyroid peroxidases antibody (TPOAb) were measured by the electrochemiluminescence immunoassay (Roche Diagnostics, Indianapolis, IN, USA) before 11:00 am on the same day. The normal range for FT3 is 3.10–6.8 pmol/L, FT4 is 10–23 pmol/L, TSH is 0.27–4.20 mIU/L, TgAb is 0–115 IU/L, TPOAb is 0–34 IU/L. The serum levels of these indicators of thyroid function are regarded abnormally high or low if they are higher or lower than the normal range, and those in the normal range are considered normal.

### Statistical analysis

Data analysis was performed using the Statistical Package for Social Sciences (SPSS version 22). We tested the normal distribution of the data using the Kolmogorov–Smirnov (KS) test. Continuous variables were expressed as mean ± standard deviation (SD) and categorical variables as frequencies and proportions (%). Independent t-test was applied for normally distributed variables. Mann–Whitney U test and Kruskal–Wallis H test were applied for non-normally distributed variables. Binary logistic regression was conducted using the enter method in order to examine which factors had a significant effect on suicide attempts in young female and male MDA patients. Variables that differed significantly between young MDA patients with and without suicide attempts were included in the binary logistic regression analyses to identify predictors of suicide attempts not only in all patients as a whole, but also in female and male patients, respectively. All comparisons between the two groups were two-sided, with a significance level of 5%.

## Results

### Prevalence and gender differences in suicide attempts between the young MDA and MDNA groups

The prevalence of suicide attempts was significantly higher in the MDA group than in the MDNA group [31.3% (146/467) in the MDA group and 7.3% (33/450) in the MDNA group; χ^2^ = 83.537, *p* < 0.001]. In the young MDA group, the rate of suicide attempts was 31.1% (51/164) in male patients compared with 31.4% (95/303) in female patients, without significant difference between male and female patients (χ^2^ = 0.003, *p* = 0.955).

In the young MDNA group, the proportion of suicide attempts was 7% (13/187) in male patients compared to 7.6% (20/263) in female patients, without significant difference between male and female patients (χ^2^ = 0.069, *p* = 0.794).

### Demographic and clinical characteristics of young MDA patients with and without suicide attempts

As shown in Table 1, there were significant differences between the two groups in terms of age (*p* = 0.001), education (*p* = 0.003), age at onset (*p* = 0.001) and duration of illness (*p* = 0.037). MDA patients with suicide attempts were older, had a later age of onset and a longer duration of illness compared to MDA patients without suicide attempts (all *p* < 0.05). In addition, MDA patients with suicide attempts had significantly higher scores on the HAMD-17, HAMA, PANSS positive symptom subscale and CGI-S than MDA patients without suicide attempts (all *p* < 0.001).

**Table 1:** Demographic and clinical characteristics of young MDA patients with and without suicide attempts.

	MDA without suicide attempts group (n = 321)	MDA with suicide attempts group (n = 146)	t/χ^2^/z	*p*
Age (years, Mean ± SD)	24.10 ± 5.35	25.92 ± 5.53	− 3.345	0.001
Gender—male, n (%)	113 (35.2%)	51 (34.9%)	0.003	0.955
Education				
Junior high school or below, n (%)	16 (5%)	17 (11.6%)	14.053	0.003
Senior high school, n (%)	164 (51.1%)	59 (40.4%)		
University degree, n (%)	122 (38%)	52 (35.6%)		
Postgraduate degree, n (%)	19 (5.9%)	18 (12.3%)		
Marital status				
Single, n (%)	183 (57%)	70 (47.9%)	3.321	0.068
Married, n (%)	138 (43%)	76 (52.1%)		
BMI (kg/m^2^, Mean ± SD)	24.39 ± 1.79	24.25 ± 2.30	0.621	0.535
Age of onset (years, Mean ± SD)	24.03 ± 5.30	25.79 ± 5.47	− 3.266	0.001
Disease duration (months, Mean ± SD)	4.74 ± 3.04	5.73 ± 4.03	− 2.085	0.037
HAMD-17 (Mean ± SD)	31.08 ± 2.61	32.64 ± 2.69	− 5.408	< 0.001
HAMA (Mean ± SD)	22.88 ± 1.89	24.35 ± 2.99	− 5.099	< 0.001
PANSS positive symptom subscale (Mean ± SD)	9.57 ± 4.67	11.76 ± 6.54	− 3.729	< 0.001
CGI-S (Mean ± SD)	6.03 ± 0.76	6.55 ± 0.69	− 7.007	< 0.001

*MDA* major depressive disorder patients with comorbid anxiety, *BMI* body mass index, *HAMD-17* Hamilton depression rating scale-17 items, *HAMA* Hamilton anxiety rating scale, *PANSS* positive and negative syndrome scale, *CGI-S* clinical global impression of severity scale.

### Thyroid function indicators in young MDA patients with and without suicide Attempts

As shown in Table 2, there were significant differences in TSH (t = − 7.290, *p* < 0.001), TgAb (z = − 3.703, *p* < 0.001) and TPOAb (z = − 3.378, *p* = 0.001) levels between young MDA patients with and without suicide attempts. Moreover, the levels of TSH, TgAb and TPOAb were higher in MDA patients with suicide attempts compared to those without suicide attempts (all *p* < 0.01). The proportion of patients with abnormally increased TSH, TgAb and TPOAb in the MDA patients with suicide attempts was significantly higher than that in the MDA patients without suicide attempts (all *p* < 0.001). We analyzed the thyroid function parameters between patients who made suicide attempts, and those who did not, in the MDNA group. We also found that there were significant differences in TSH (z = − 3.524, *p* < 0.001), FT3 (z = − 2.441, *p* = 0.015), TgAb (z = − 2.756, *p* = 0.006) and TPOAb levels (z = − 3.416, *p* = 0.001) between young MDNA patients with and without suicide attempts. Moreover, the levels of TSH, TgAb and TPOAb were higher in MDNA patients with suicide attempts compared to those without suicide attempts (all *p* < 0.01) and the level of FT3 was lower in MDNA patients with suicide attempts compared to those without suicide attempts (*p* < 0.05).

**Table 2:** Comparison of thyroid function indicators in young MDA patients with and without suicide attempts.

	MDA without suicide attempts group (n = 321)	MDA with suicide attempts group (n = 146)	t/χ^2^/z	*p*
TSH (mIU/mL, Mean ± SD)	4.73 ± 2.55	6.68 ± 2.95	− 7.290	< 0.001
TSH Decrease (< 0.27 mIU)	0	0	13.158	< 0.001
TSH Normal (0.27–4.20 mIU/L)	140 (43.6%)	38 (26%)		
TSH Increase (> 4.20 mIU/L)	181 (56.4%)	108 (74%)		
FT3 (pmol/L, Mean ± SD)	4.97 ± 0.69	4.90 ± 0.81	0.938	0.349
FT3Decrease (< 3.10 pmol/L)	0	0	2.203	0.138
FT3Normal (3.10–6.8 pmol/L)	321 (100%)	145 (99.3%)		
FT3Increase (> 6.8 pmol/L)	0	1 (0.7%)		
FT4 (pmol/L, Mean ± SD)	16.65 ± 3.03	16.95 ± 3.33	− 0.862	0.389
FT4Decrease (< 10 pmol/L)	0	0	0.914	0.339
FT4Normal (10–23 pmol/L)	319 (99.4%)	146 (100%)		
FT4Increase (> 23 pmol/L)	2 (0.6%)	0		
TgAb (IU/L, Mean ± SD)	92.08 ± 244.84	137.86 ± 290.37	− 3.703	< 0.001
TgAb Normal (0–115 IU/L)	270 (84.1%)	102 (69.9%)	12.574	< 0.001
TgAb Increase (> 115 IU/L)	51 (15.9)	44 (30.1%)		
TPOAb (IU/L, Mean ± SD)	52.10 ± 98.02	133.84 ± 236.47	− 3.378	0.001
TPOAb Normal (0–34 IU/L)	247 (76.9%)	84 (57.5%)	18.322	< 0.001
TPOAb Increase (> 34 IU/L)	74 (23.1%)	62 (42.5%)		

*MDA* major depressive disorder patients with comorbid anxiety, *TSH* thyroid stimulating hormone, *FT3* free triiodothyronine, *FT4* free thyroxine, *TgAb* anti-thyroglobulinand, *TPOAb* thyroid peroxidases antibody.

### Risk factors and sex differences of suicide attempts in young MDA patients

We analyzed sex differences in thyroid parameters in the male MDA patients with and without suicide attempts, and in the female MDA patients with and without suicide attempts. We found significant differences in TSH (H = 44.623, *p* < 0.001), TgAb (H = 14.296, *p* = 0.003) and TPOAb (H = 12.558, *p* = 0.006) levels among the four groups. Although there was no significant sex difference in thyroid parameters between male and female patients with or without suicide attempts (all *p* > 0.05), the thyroid parameters showed different characteristics in male and female patients with or without suicide attempts. TSH (H = − 84.246, *p* < 0.001) and TPOAb levels (H = − 64.780, *p* = 0.027) were significantly different between suicide attempters and non-suicide attempters in male patients, while TSH (H = − 92,836, *p* < 0.001) and TgAb (H = − 52.788, *p* = 0.010) levels were significantly different between suicide attempters and non-suicide attempters in female patients.

Variables that were significantly different between young MDA patients with and without suicide attempts were included in binary logistic regression analyses to identify predictors of suicide attempts not only in the whole group, but also in female and male patients, respectively. As shown in Table 3, HAMA score (OR 1.223, 95% CI 1.084–1.380, *p* = 0.001), CGI-S score (OR 1.892, 95% CI 1.311–2.731, *p* = 0.001), TSH (OR 1.162, 95% CI 1.052–1.283, *p* = 0.003) and TPOAb levels (OR 1.003, 95% CI 1.002–1.005, *p* < 0.001) were independently associated with suicide attempts in young MDA patients.

**Table 3:** Risk factors for suicide attempts in young MDA patients.

Variable	B	S.E	Wald	*p*	OR (95% CI)
Age	− 0.072	0.447	0.026	0.871	0.930 (0.387–2.235)
Education level	0.027	0.153	0.030	0.861	1.027 (0.761–1.386)
Age of onset	0.122	0.447	0.074	0.785	1.130 (0.470–2.714)
Disease duration	0.068	0.047	2.091	0.148	1.070 (0.976–1.173)
HAMD-17	0.018	0.060	0.088	0.767	1.018 (0.906–1.144)
HAMA	0.201	0.062	10.651	0.001	1.223 (1.084–1.380)
PANSS positive symptom	− 0.041	0.028	2.064	0.151	0.960 (0.908–1.015)
CGI-S	0.638	0.187	11.598	0.001	1.892 (1.311–2.731)
TSH	0.150	0.051	8.822	0.003	1.162 (1.052–1.283)
TPOAb	0.003	0.001	13.084	< 0.001	1.003 (1.002–1.005)
TgAb	− 0.001	0.000	1.218	0.270	0.999 (0.998–1.000)

*HAMD-17* Hamilton depression rating scale-17 items, *HAMA* Hamilton anxiety rating scale, *PANSS* positive and negative syndrome scale, *CGI-S* clinical global impression of severity scale, *TSH* thyroid stimulating hormone, *TPOAb* thyroid peroxidases antibody, *TgAb* anti-thyroglobulinand.

As shown in Tables 4 and 5, there were some differences in risk factors for suicide attempts between male and female patients. For male patients, levels of TSH (OR 1.262, 95% CI 1.062–1.499, *p* = 0.008) and TPOAb (OR 1.004, 95% CI 1.000–1.007, *p* = 0.031) were independently associated with suicide attempts. For female patients, HAMA score (OR 1.326, 95% CI 1.135–1.550, *p* < 0.001), PANSS positive symptom score (OR 0.921, 95% CI 0.857–0.990, *p* = 0.026), CGI-S score (OR 2.446, 95% CI 1.487–4.024, *p* < 0.001) and TPOAb levels (OR 1.003, 95% CI 1.001–1.006, *p* = 0.002) were independently associated with suicide attempts.

**Table 4:** Risk factors for suicide attempts in young male MDA patients.

Variable	B	S.E	Wald	*p*	OR (95% CI)
Age	− 0.375	0.586	0.409	0.523	0.687 (0.218–2.169)
Education level	0.196	0.303	0.420	0.517	1.217 (0.672–2.201)
Age of onset	0.394	0.582	0.457	0.499	1.483 (0.473–4.644)
Disease duration	0.087	0.072	1.463	0.226	1.090 (0.948–1.255)
HAMD-17	− 0.104	0.099	1.102	0.294	0.902 (0.743–1.094)
HAMA	0.125	0.118	1.109	0.292	1.133 (0.898–1.428)
PANSS positive symptom	0.003	0.051	0.004	0.948	1.003 (0.908–1.108)
CGI-S	0.311	0.302	1.062	0.303	1.365 (0.755–2.469)
TSH	0.233	0.088	7.016	0.008	1.262 (1.062–1.499)
TPOAb	0.004	0.002	4.631	0.031	1.004 (1.000–1.007)
TgAb	− 0.001	0.001	1.066	0.302	0.999 (0.996–1.001)

*HAMD-17* Hamilton depression rating scale-17 items, *HAMA* Hamilton anxiety rating scale, *PANSS* positive and negative syndrome scale, *CGI-S* clinical global impression of severity scale, *TSH* thyroid stimulating hormone, *TPOAb* thyroid peroxidases antibody, *TgAb* anti-thyroglobulinand.

**Table 5:** Risk factors for suicide attempts in young female MDA patients.

Variable	B	S.E	Wald	*p*	OR (95% CI)
Age	0.448	0.722	0.386	0.535	1.566 (0.380–6.442)
Education level	− 0.016	0.185	0.007	0.932	0.984 (0.685–1.414)
Age of onset	− 0.383	0.724	0.280	0.597	0.682 (0.165–2.819)
Disease duration	0.030	0.066	0.209	0.647	1.031 (0.905–1.174)
HAMD-17	0.122	0.079	2.391	0.122	1.130 (0.968–1.320)
HAMA	0.282	0.079	12.656	< 0.001	1.326 (1.135–1.550)
PANSS positive symptom	− 0.082	0.037	4.987	0.026	0.921 (0.857–0.990)
CGI-S	0.895	0.254	12.408	< 0.001	2.446 (1.487–4.024)
TSH	0.097	0.063	2.371	0.124	1.102 (0.974–1.248)
TPOAb	0.003	0.001	9.237	0.002	1.003 (1.001–1.006)
TgAb	0.000	0.001	0.165	0.685	1.000 (0.999–1.001)

*HAMD-17* Hamilton depression rating scale-17 items, *HAMA* Hamilton anxiety rating scale, *PANSS* positive and negative syndrome scale, *CGI-S* clinical global impression of severity scale, *TSH* thyroid stimulating hormone, *TPOAb* thyroid peroxidases antibody, *TgAb* anti-thyroglobulinand.

## Discussion

To our knowledge, this is the first study to explore the prevalence of suicide attempts and the association with indicators of thyroid function, as well as their sex differences, in first-episode and drug-naïve young MDD patients with comorbid anxiety in a large-scale cross-sectional design. Our study found that (1) the prevalence of suicide attempts was 31.3% in young MDA patients, which was significantly higher than that in MDNA patients. Moreover, there were no gender differences in the prevalence between the two groups; (2) compared with MDA patients without suicide attempts, MDA patients with suicide attempts were older, had a later age of onset and longer duration of illness, and had higher HAMD-17, HAMA, PANSS positive symptom subscale, CGI-S scores, and TSH, TgAb and TPOAb levels; and (3) thyroid parameters showed different characteristics in male and female MDA patients with or without suicide attempts.

Despite being considered a serious public health problem, suicidal behavior in young adults with depression is often unrecognized and untreated^22^. In our study, the prevalence of suicide attempts was significantly higher in the MDA patients than in the MDNA group, which supports the idea that anxiety increases the risk of suicide in depressed patients^23,24^. In patients with MDD, anxiety, especially the agitation that may accompany it, increases the severity of the illness, which in turn increases the risk of suicide. Previous studies have shown that in the general population, suicide attempts are 2.31 times more frequent in patients with MDA than in patients with MDNA^25^. Our study found that in the younger population, the frequency of suicide attempts was about 4.29 times higher in MDA patients than in MDNA patients. This suggests that anxiety may have a greater impact on suicide in young depressed patients and that these patients should receive more attention and early intervention. In our study, we did not find gender differences in suicide attempts in young MDA and MDNA patients. A large number of previous studies have found gender differences in suicide among depressed patients, although the findings are not entirely consistent. In the general population of most Western countries, suicide rates are traditionally higher in males than in females, but in Asian countries, especially in China, the opposite results are usually reported^26^. For example, Ruengorn et al.^27^ found that men with depression were more likely to attempt suicide; Miret et al.^28^ demonstrated that men were a major risk factor for suicide in patients with MDD. A recent systematic review also supported that men with depression were at significantly greater risk for suicide^7^; however, another meta-analysis showed that the lifetime prevalence of suicide attempts in men was similar to that in women in China. Several factors may contribute to this inconsistency in suicide attempt rates, particularly the different sources of subjects and different cultures.

Suicide risk varies with the nature of MDD and other demographic and clinical factors^7^. In our study, we found that young MDA patients with suicide attempts were older and had a later age of onset. These findings are inconsistent with some previous studies, which found younger age and earlier age of onset to be risk factors^13,27,29^. These discrepancies may be due to the fact that we focused only on the young age group. When compared to patients of all ages in the general population, these patients appear to be younger and have an earlier age of onset. We also found that young MDA patients with suicide attempts had a longer disease duration; this finding is consistent with a previous series of studies^27,30^. Our study suggested that young MDA patients with suicide attempts had more severe symptoms, including higher levels of depression and anxiety. More importantly, these patients were more likely to have psychotic symptoms. These findings are consistent with several previous studies that have found that more severe depression^31^ and comorbid anxiety^7^ or psychotic symptoms^32,33^ are associated with suicide in depressed patients.

It is well known that thyroid dysfunction may significantly affect mental health conditions, including depression, anxiety, and suicide. Previous studies have found that, on average, 40% and 30% of patients with hypothyroidism experience depression and anxiety^34^. More importantly, thyroid dysfunction may influence the development of mood disorders. Our previous study showed that serum levels of TSH, TgAb and TPOAb were higher in MDA patients with suicide attempts compared to MDA patients without suicide attempts in general population^35^. In this study, we found higher TSH, TgAb and TPOAb levels but comparable T3 and T4 levels in young MDA patients with suicide attempts compared to those without suicide attempts. The proportion of patients with abnormally increased TSH, TgAb and TPOAb in the MDA patients with suicide attempts was significantly higher than that in the MDA patients without suicide attempts. It was consistent with our previous works on the MDD patients in general population^36^. This suggests that subclinical hypothyroidism may play an important role in suicide in these patients. Our results are partially consistent with those of some previous studies. For example, Shen et al.^36^ demonstrated higher serum TSH levels in patients with suicide attempts while others found adverse consequences^37,38^. In contrast, some other studies found significant differences in T3 or T4 levels between patients with and without suicide attempts^39^. Several factors, such as sample size, and the effects of antidepressants may contribute to these differences. There is evidence that thyroid hormones may play a role in the regulation of neurotransmitters involved in the pathogenesis of suicide such as 5-hydroxytryptamine (5-HT) and norepinephrine^9^. Duval et al. postulated that a decrease in 5-HT function triggered an increased thyrotropin-releasing hormone (TRH) secretion that secondarily normalized 5-HT neurotransmission and also maintained normal thyroid hormone levels but this compensatory mechanism might be not effective in depressed patients with a history of suicidal behavior^38^. TRH from the hypothalamus stimulates the synthesis and release of TSH and from the pituitary thyrotrophs as well as that of prolactin (PRL) from the lactotrophs. Duval et al. found that depressed patients with suicidal behavior in early remission showed normal thyroid and lactotroph axes activity but in current suicidal depressed patients, these axes might have deficiencies^38^. We hypothesize that the slowing down of thyroid function is a gradual process that may start with an increase in TSH and gradually affect T3 and T4 levels due to the compensatory function of the thyroid gland. In young MDD patients, this change in thyroid function may still be in its early stages, so they mainly present with abnormal TSH levels. Our study found that young MDA patients with suicide attempts had more severe depression, anxiety and psychotic symptoms. It is possible that elevated TSH levels may increase the severity of anxiety, depression and psychotic symptoms through mechanisms that are still unknown, thereby increasing the risk of suicide in MDD patients^36^. Interestingly, the result that the level of FT3 was lower in MDNA patients with suicide attempts compared to those without suicide attempt was not found in MDA patients. We hypothesized that this phenomenon was related to the impact of anxiety on thyroid function which needed further research. We also found higher levels of TgAb and TPOAb, which are autoimmune thyroiditis antibodies, in MDA patients with suicide attempts. Increased TgAb and TPOAb levels indicate that the thyroid tissue is in an active state of immune inflammation. This result suggests that suicide may be associated with autoimmune hypothyroidism. In fact, much evidence suggests that the occurrence of suicide, depression and other affective disorders have been previously associated with autoimmune diseases such as Hashimoto's thyroiditis^40–42^. Autoimmune thyroid disease itself increases the risk of suicide in general population^43^. Dysregulation of the immune system has been shown to be involved in the pathophysiology of depression^44–46^, anxiety^47,48^, and suicide^49–51^. Patients with immune activation are at higher risk of suicide attempts, which may be due to increased neurotoxicity due to inflammation and nitro-oxidative stress^51^. Previous studies also indicated that MDD patients with higher TPOAb levels had more severe depression and anxiety symptoms, which might elevate the risk of suicide attempt^35^.

We didn’t find significant sex difference in thyroid parameters between male and female patients with or without suicide attempts, but the thyroid parameters showed different characteristics in male and female patients with or without suicide attempts. Feng et al. found that MDD patients with suicide attempts presented with an elevated blood concentration of TSH, TG-Ab and TPO-Ab, but not FT3 and FT4 without gender differences^52^. Our study supported these findings. Further, we found some differences in risk factors for suicide attempts between male and female patients. The indicators of thyroid function which could predict suicide attempts in MDA patients had sex differences. While TPOAb levels were independently associated with suicide attempts in both male and female patients, only TSH levels were independently associated with suicide attempts in male patients. These findings suggest that although both male and female patients have active immune inflammatory thyroid tissue, subclinical hypothyroidism may be present only in male patients or may have a differential impact on suicide depending on sex. This suggests that young male MDA patients with suicide attempts may have more severe thyroid dysfunction than female MDA patients. Our results are inconsistent with some previous studies. For example, Bartova et al.^53^ found a higher rate of somatic comorbidities (including thyroid dysfunction) in female MDD patients. Possible reasons for this inconsistency are that we recruited only young MDD patients and that these patients were comorbid with anxiety; these factors may have different effects on thyroid function by sex. In our study, we found anxiety and psychotic symptoms along with disease severity were risk factors for suicide attempts in female patients. We hypothesized that thyroid dysfunction may have direct and indirect effects on suicide in depressed patients. For male patients, the direct effect may be more pronounced, whereas for female patients, thyroid dysfunction may indirectly increase the risk of suicide partly through some clinical symptoms (e.g., anxiety and psychotic symptoms).

The present study has several limitations. First, this was a cross-sectional study and it was not possible to determine the causal relationship between thyroid function indicators and suicide attempts. Our findings need to be confirmed by a prospective cohort study. Second, we collected information about suicide through interviews rather than using a structured instrument. Quantitative assessment tools need to be used in future studies. Third, the participants recruited in our study were from the Chinese Han population. Therefore, our findings should be validated in other ethnically diverse populations. Fourth, the best way to diagnose mental disorders is through psychiatric interviews. In this study, we determined anxiety symptoms only by HAMA, which is one of the methodological limitations that should be remedied in future studies.

## Conclusion

In summary, our study found that the prevalence of suicide attempts was higher in young MDA patients than in MDNA patients, and there were no sex differences. This suggests that for the young population, male and female MDA patients face the same suicide risk and should be given adequate suicide assessment and early intervention. Patients with suicide attempts had thyroid dysfunction compared to young MDA patients without suicide attempts. In addition, the indicators of thyroid function which could predict suicide attempts in MDA patients had sex differences. We found that TPOAb levels were independently associated with suicide attempts in both male and female patients, whereas TSH levels were independently associated with suicide attempts in male patients only. In addition, anxiety levels, psychiatric symptoms, and disease severity were risk factors for suicide attempts only in female patients. However, because of the limitations mentioned above, future studies will need to use a longitudinal design to confirm the findings in this study.

## Data Availability

The data that support the findings of this study are available on request from the corresponding author upon reasonable request.
